# Influence of *Chrysanthemum morifolium*-maize intercropping pattern on yield, quality, soil condition, and rhizosphere soil microbial communities of *C. morifolium*

**DOI:** 10.3389/fpls.2024.1383477

**Published:** 2024-04-24

**Authors:** Zhiyuan Liao, Qiaohuan Chen, Jinxin Li, Lu Wei, Jialiang Wu, Xiao Wang, Qi Liu, Yuhuan Miao, Dahui Liu

**Affiliations:** ^1^ School of Pharmacy, Hubei University of Chinese Medicine, Wuhan, China; ^2^ Hubei shizhen Laboratory, Hubei University of Chinese Medicine, Wuhan, China

**Keywords:** *Chrysanthemum morifolium*, maize, intercropping, yield, quality, soil condition, rhizosphere soil microbial communities

## Abstract

**Introduction:**

*Chrysanthemum morifolium* Ramat. is a perennial herb in the Compositae family, often employed in traditional Chinese medicine due to its medicinal value. The planting of *C. morifolium* faces the challenges of continuous cropping, and intercropping is able to somewhat overcome the obstacles of continuous cropping.

**Methods:**

In our study, we designed two different *C. morifolium*-maize intercropping patterns, including *C. morifolium*-maize narrow-wide row planting (IS) and *C. morifolium*-maize middle row planting (IM). Compared with monoculture, the agronomic traits, yield, active ingredients, soil physicochemical properties, soil enzyme activities, and rhizosphere soil microbial communities of *C. morifolium* and maize were measured under the two *C. morifolium*-maize intercropping patterns.

**Results:**

The findings indicated that (1) Intercropping elevated the agronomic traits, yield, and active ingredients of *C. morifolium*, especially in *C. morifolium*-maize narrow-wide row planting pattern, which indicating that interspecific distance played an important role in intercropping system; (2) Intercropping enhanced soil physicochemical properties and enzyme activities of *C. morifolium* and maize; (3) Intercropping altered rhizosphere soil microbial communities of *C. morifolium* and maize, making microbial interrelationships more complex. (4) Intercropping could recruit a large number of beneficial microorganisms enrich in the soil, including *Bacillus*, *Sphingomonas*, *Burkholderia-Caballeronia-Paraburkholderia*, *Chaetomium*, and *Ceratorhiza*, which may increase the content of AN, NN, AvK, ExCa, AvCu, AvZn and other nutrients in soil and promoted the growth and quality of *C. morifolium.*

**Discussion:**

In summary, intercropping with maize could promote the accumulation of beneficial microorganisms in the soil, thus improving the overall growing environment, and finally realizing the growth and improvement of *C. morifolium*.

## Introduction

In recent years, monoculture has been widely used because of its ease of planting, field management and mechanization. However, monoculture deprived the soil of nutrients, limiting its ability to support healthy plant growth in the long term (Wolińska et al., 2015). Therefore, monoculture usually improved yields by increasing fertilizer application, which in turn disrupt the natural composition of the soil, leading to further loss of nutrients from the soil (Wolińska et al., 2022). Moreover, continuous monocultures limited the diversity of soil microbial species (Yusuf et al., 2009), disrupted the ecological stability of the soil microbial community (Misra et al., 2019), and resulted in the spread of pests and diseases (Brooker et al., 2015). Many medicinal plants and crops suffered from these negative effects (Arafat et al., 2019; Li et al., 2019; Liu et al., 2020; Gong et al., 2022).

To overcome these problems, intercropping can be used as an alternative. Intercropping refers to the simultaneous planting of several (two or more) crops on the same land (Zhao et al., 2018; Stomph et al., 2020), acting as a sustainable approach applied to modern agricultural production systems globally (Zhu et al., 2015; Zaeem et al., 2019). Compared to monoculture, intercropping could better utilize light, water, and nutrient resources (Willey, 1990; Ren et al., 2017), improve soil quality (Cong et al., 2015), limit soil erosion (Chen et al., 2010; Kurothe et al., 2014), enhance crop yield (Gronle et al., 2015; Gou et al., 2016; Latati et al., 2016), and increase land use efficiency (Wang et al., 2015). Intercropping usually has a profound effect on soil, the green garlic-cucumber intercropping has been proved to regulate the soil micro-ecological environment by improving the content of AvP, AvK, and other nutrients (Xiao et al., 2019). Prior studies also found that intercropping could help enhance the crop yield and quality (Liu et al., 2021). Moreover, intercropping can change the microbial communities to improve soil condition, microorganisms are directly related to the decomposition of organic matter and the transformation of mineral elements in the soil, which in turn affects the growth and quality of crops (Anderson, 2003). Lily-maize intercropping could produce beneficial effects on lily through influencing the diversity and structure of the bacterial community and increasing the relative abundance of beneficial bacteria in the lily rhizosphere soil (Zhou et al., 2018). Therefore, intercropping may be an effective way to solve the dilemma of continuous monoculture.

*Chrysanthemum morifolium* Ramat. acts as a medicinal plant member of the Compositae family, which originated in China has been used for over 3000 years (Yuan et al., 2020), and is commonly employed as a traditional Chinese medicinal drink (Wang et al., 2019). The primary active ingredients in *C. morifolium* include caffeoylquinic acids such as 3-O-caffeoylquinic acid, 3,5-dicaffeoylquinic acid, as well as luteolin-7-O-glucoside and other flavonoids (Ranxin et al., 2004; Kim and Lee, 2005; Xie et al., 2012). Currently, in response to the increasing demand for *C. morifolium* for medicinal uses, cultivation has expanded dramatically. *C. morifolium* has typically been planted in long-term continuous monocultures. However, numerous studies have indicated that *C. morifolium* continuous monocultures led to massive reproduction of pathogens, soil nutrients imbalance, changing in soil microbial community, and the destruction of soil structure (Wang et al., 2011; Liu et al., 2012).

Thus, in order to solve the current problems in the cultivation of *C. morifolium*, we proposed the *C. morifolium*-maize intercropping system. Several studies have demonstrated that maize is suitable for intercropping with medicinal plants and crops (Jiao et al., 2021; Noushahi et al., 2021; Zhang et al., 2021; Peng et al., 2022). Maize promotes the sustainable productivity of intercropping plants by increasing beneficial soil microorganisms, altering the microbial structure, improving microbial abundance, inhibiting disease occurrence, and promoting nitrogen absorption (Fan et al., 2019; Chang et al., 2020). However, intercropping between *C. morifolium* and maize has been rarely reported.

To investigate the advantages of *C. morifolium-*maize intercropping, two different patterns were employed to study the effect of intercropping on agronomic traits, yield, and active ingredients of *C. morifolium*, as well as the effects on soil physicochemical properties, soil enzyme activities, and rhizosphere soil microbial communities. This study will lay a foundation for the promotion and application of *C. morifolium-*maize intercropping patterns in *C. morifolium* production.

## Materials and methods

### Experimental location

The experimental site was located at Hubei University of Chinese Medicine, Wuhan City, Hubei Province, China (30° 37’ N, 114° 37’ E, altitude 30 m), in the North subtropical monsoon climate region. Before the experiment, the following chemical properties of the yellow-brown soil in the test field were determined: pH 6.80, soil organic matter (SOM) 20.72 g·kg^-1^, total nitrogen (TN) 1.07 g·kg^-1^, total phosphorus (TP) 0.77 g·kg^-1^, and total potassium (TK) 22.37 g·kg^-1^.

### Experimental design and field management

**Figure 1:**
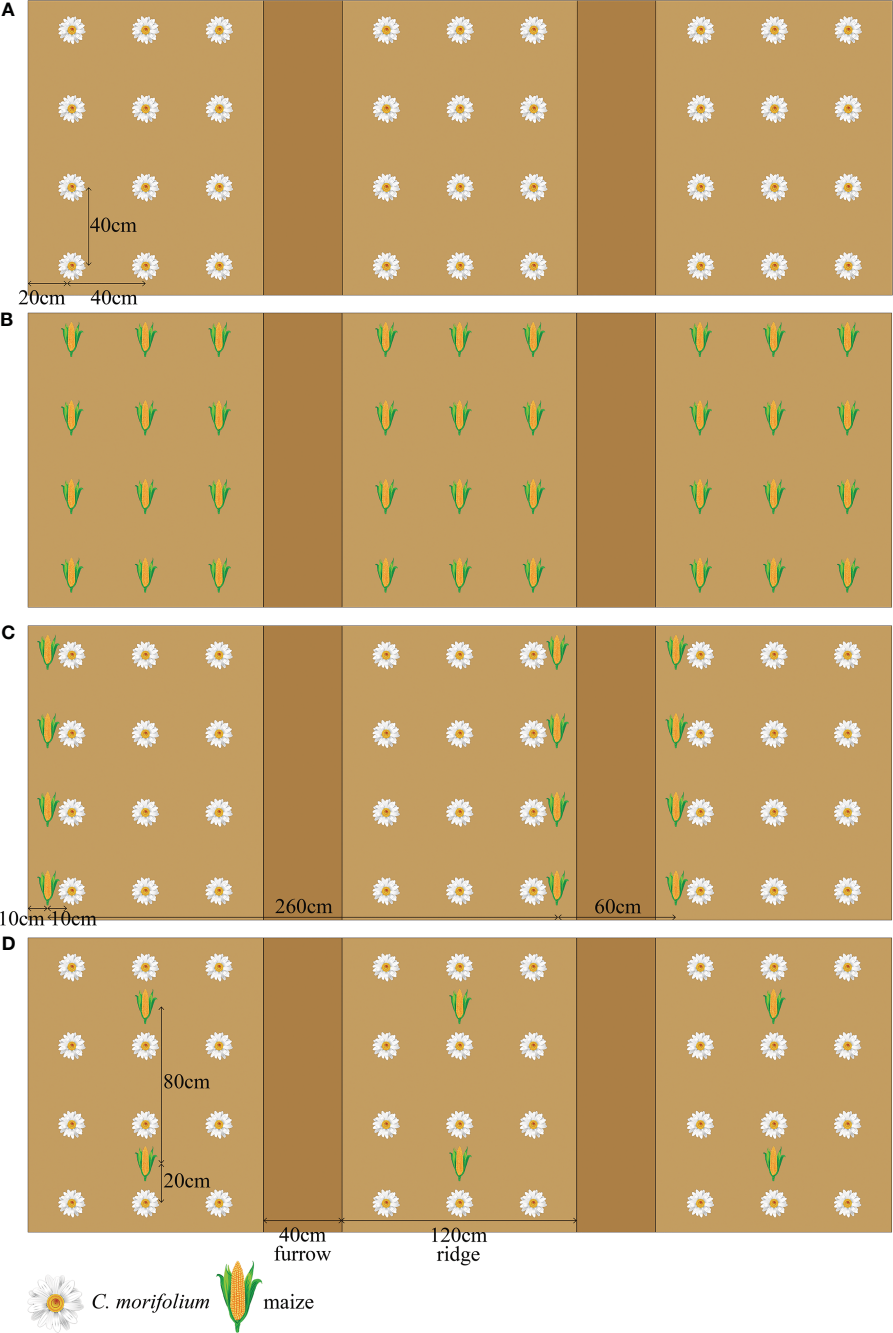
Schematic diagram of *C*. *morifolium-*maize intercropping and monoculture treatments. **(A)** monoculture *C*. *morifolium* (MC); **(B)** monoculture maize (MM); **(C)** *C*. *morifolium*-maize intercropping, *C*. *morifolium*-maize narrow-wide row planting (IS, *C*. *morifolium* and maize of IS known as ISC and ISM, respectively); **(D)** *C*. *morifolium*-maize intercropping, *C*. *morifolium*-maize middle row planting (IM, *C*. *morifolium* and maize of IM known as IMC and IMM, respectively).

This experiment was conducted in May 2022 using three replicates of four treatments in a randomized block design. Monoculture *C. morifolium* (MC) and monoculture maize (MM) were established as control, the transverse and longitudinal distance between each *C. morifolium* or maize and its surrounding *C. morifolium* or maize was 40 cm × 40 cm (**Figures 1A, B**). Two intercropping patterns of *C. morifolium-*maize intercropping were established as follows: *C. morifolium-*maize narrow-wide row planting (IS), i.e., the relay intercropping combination of two crop strips with a total width of 320 cm, composed of two rows of maize and six rows of *C. morifolium* with 60 cm row width for maize, 40 cm or 80 cm row width for *C. morifolium*, and 10 cm spacing between the adjacent rows of maize and *C. morifolium* (*C. morifolium* and maize of IS were known as ISC and ISM, respectively) (**Figure 1C**); *C. morifolium-*maize middle row planting (IM), i.e., maize was planted between the middle row of *C. morifolium*, 80* cm* plant spacing for maize, 40 cm plant spacing for *C. morifolium*, and 20 cm spacing between the adjacent plants of maize and *C. morifolium* (*C. morifolium* and maize of IM were known as IMC and IMM, respectively) (**Figure 1D**). In two different intercropping patterns, the transverse and longitudinal distance between each *C. morifolium* and its surrounding *C. morifolium* was 40 cm × 40 cm (**Figures 1C, D**). *C. morifolium* and maize were planted on ridges (20 cm high); each ridge was 120 cm wide and separated by a 40 cm wide furrow. Basal fertilizers were administered to the soils prior to the planting of the *C. morifolium* and maize. The organic fertilizer [organic matter ≥ 70%, N-P_2_O_5_-K_2_O ≥ 6% (2-2-2)] and compound fertilizer [N-P_2_O_5_-K_2_O ≥ 40% (20-8-12)] were administered at rates of 7500 kg·ha^-1^ and 750 kg·ha^-1^ as basal fertilizers, respectively. All field management practices were consistent across treatments, including intertillage, weeding, watering, drainage, disease control, and topping.

### Sampling of rhizosphere soil

Each rhizosphere soil sample was obtained from the *C. morifolium* and maize rhizosphere of each intercropping and monoculture pattern at the flowering stage of *C. morifolium* (November 2022) and at the mature stage of maize (September 2022). The rhizosphere soils from five randomly selected *C. morifolium* and maize plants were obtained in each replicate of each treatment as one biological replicate, with three biological replicates performed in each treatment. A total of 18 rhizosphere soil samples were acquired. After removing non-soil particles, crushing, and passing through a 2-mm sieve, the rhizosphere soil was separated into two segments. One segment was stored in a -80°C freezer and used to analyze soil microbial community structure and diversity detection; the second segment was dried at room temperature and pulverized into a powder to detect soil physicochemical properties and enzyme activities.

### Determination of agronomic traits and yield of *C. morifolium* and maize

Fifteen *C. morifolium* and maize plants were randomly chosen from each experimental treatment to examine agronomic traits and yield. Measurements of *C. morifolium* included plant height, stem diameter, number of first branches, number of second branches, leaf length, leaf width, flower outside diameter, flower inside diameter, number of flowers per plant, 100-fresh flower weight, fresh flower weight per plant, dry flower weight per plant. Measurements for maize included grain weight per plant. The land equivalent ratio (LER) represented the total land area of crops required to achieve the same yield as the intercrops: LER = Y_ic_/Y_mc_ + Y_im_/Y_mm_. Y_ic_ and Y_mc_ represented the yield of intercropping and monoculture *C. morifolium*, and Y_im_ and Y_mm_ were the yields of intercropping and monoculture maize, respectively. The yields of *C. morifolium* and maize were converted by multiplying the fresh flower weight and grain weight per plant by planting density, respectively. Intercropping systems exhibit yield advantages when LER > 1, while LER < 1 exhibits yield disadvantages (Mead and Willey, 1980).

### Determination of active ingredients of *C. morifolium*

3-O-caffeoylquinic acid, rutin, luteolin-7-O-glucoside, 3,4-dicaffeoylquinic acid, 3,5-dicaffeoylquinic acid, and 4,5-dicaffeoylquinic acid were obtained to use as a control from China National Institute for the Control of Pharmaceuticals and Biological Products (Beijing, China). Sample solutions were prepared according to the method in Pharmacopoeia of the People's Republic of China (2020 Edition), Part 1, medicinal materials and cut crude drugs, *Chrysanthemum*. The contents of the primary active ingredients in *C. morifolium* were determined using a Shimadzu LC-2030C 3D PLUS high-performance liquid chromatography (HPLC) instrument alongside an Agilent ZORNAX SB-C18 column (4.6 mm × 250 mm, 5 μm). A binary solvent system of A) 0.1% phosphoric acid in water and B) acetonitrile (v/v) was utilized with a 0.8 mL·min^-1^ flow rate. The injection volume was 20 μL, and gradient elution including 0-8 minutes, 16% B; 8-13 minutes, 16%-20% B; 13-30 minutes, 20% B; 30-35 minutes, 20%-25% B; 35-60 minutes, 25%-30% B; 60-70 minutes, 30-60% B; 70-80 minutes, 60% B. The detection ultraviolet wavelength was established at 348 nm, and the column temperature was 30°C (Wang et al., 2022).

### Determination of soil physicochemical properties and enzyme activities of *C. morifolium* and maize

Soil pH was identified using the potentiometric method in which the soil-to-water ratio was 1:2.5. Soil organic matter (SOM) was determined using the potassium dichromate volumetric method. The total nitrogen (TN) was determined by the Kjeldahl method. Total phosphorus (TP) was determined through the molybdenum antimony colorimetry method. Total potassium (TK), exchangeable calcium (ExCa), exchangeable magnesium (ExMg), available ferrum (AvFe), available manganese (AvMn), and available zinc (AvZn) were determined using the flame spectrophotometry method. Available cuprum (AvCu) was determined through the graphite furnace spectrophotometry method. Available boron (AvB) was determined by the Azomethime-H colorimetry method (Bao, 2000). Ammonium nitrogen (AN), nitrate nitrogen (NN), available phosphorus (AvP), and available potassium (AvK) were determined using assay kits produced by Beijing Solarbio Science & Technology Co., Ltd., according to the manufacturer’s instructions. Soil urease (S-UE) activity was determined by the phenolsodium hypochlorite colorimetric method. Soil sucrase (S-SC) activity was determined using the 3,5-dinitrosalicylic acid colorimetric method. Soil acid phosphatase (S-ACP) activity was determined by the disodium phenyl phosphate colorimetric method. Soil catalase (S-CAT) activity was determined using the potassium permanganate titration method (Guan et al., 1986).

### Soil DNA extraction, PCR amplification, and Illumina Miseq sequencing

Total DNA was extracted from the microbial community according to the E.Z.N.A.^®^ soil DNA kit (Omega Bio-tek, Norcross, GA, U.S.) instructions, and DNA extraction quality was measured using 1% agarose gel electrophoresis. DNA concentration and purity were determined using a NanoDrop2000. The primers 338F (5’-ACTCCTACGGGAGGCAGCAG-3’) and 806R (5’-GGACTACHVGGGTWTCTAAT-3’) were used for PCR amplification of the V3-V4 variable region of the 16S rRNA gene and ITS1F (5’-CTTGGTCATTTAGAGGAAGTAA-3’) and ITS2R (5’-GCTGCGTTCTTCATCGATGC-3’) were used for PCR amplification of the I1-I2 variable region of the ITS rRNA gene. The amplification procedure was as follows: pre-denaturation at 95°C for 3 minutes, followed by 27 cycles of denaturation at 95°C for 30 seconds, annealing at 55°C for 30 seconds, extension at 72°C for 30 seconds, followed by a final extension at 72°C for 10 minutes, and finally storage at 4°C (PCR instrument: ABI GeneAmp^®^ 9700). The 20 μL PCR reaction system was made up of: 5×TransStart FastPfu buffer, 4 μL; 2.5 mM dNTPs, 2 μL; each primer (5 μM), 0.8 μL; TransStart FastPfu DNA polymerase, 0.4 μL; template DNA, 10 ng; ddH_2_O, to the final volume of 20 μL. There were three replicates performed per sample. PCR products from the same sample were combined and recovered using 2% agarose gel electrophoresis. The recovered products were purified using an AxyPrep DNA Gel Extraction Kit (Axygen Biosciences, Union City, CA, USA) and quantified by a Quantus™ Fluorometer (Promega, USA). Sequencing was conducted using an Illumina Miseq PE300 platform (Shanghai Meiji Biomedical Technology Co., LTD.). Raw data was uploaded to the NCBI SRA database (serial number: SRR26142235-SRR26142252, SRR26450172-SRR26450189).

### Data processing statistical analysis

Fastp (https://github.com/OpenGene/fastp, version 0.20.0) software was utilized to perform quality control on the original sequencing sequence (Chen et al., 2018). FLASH (http://www.cbcb.umd.edu/software/flash, version 1.2.7) software was employed for Mosaic (Magoč and Salzberg, 2011): (1) The base of reads with a mass value below 20 at the tail end was removed, and a 50 bp window was established. (2) According to the overlap between PE reads, pairs of reads were merged into a sequence with a minimum overlap length of 10 bp. (3) The maximum mismatch ratio allowed in the overlap area of the splicing sequence was 0.2, and the non-conforming sequence was screened. (4) The samples were differentiated based on the barcode and primers at both ends of the sequence, and the sequence direction was adjusted. The allowable mismatch number of the barcode was 0, and the maximum mismatch number of the primer was 2. Using UPARSE software (http://drive5.com/uparse/, version 7.1) (Edgar, 2013), according to 97% OTU on the sequence of similarity clustering and eliminate chimeras (Stackebrandt and Goebel, 1994; Edgar, 2013). Species classification annotation was conducted on each sequence using the RDP classifier (http://rdp.cme.msu.edu/, version 2.2) (Wang et al., 2007). The sequence was compared using the Silva database (Release138 http://www.arb-silva.de) of the 16S rRNA gene and the Unite database (Release 8.0 http://unite.ut.ee/index.php) of the ITS rRNA gene, and the comparison threshold was established as 70%.

### Statistical analysis

Microsoft Excel 2010 software and SPSS 26.0 software were employed for statistical data analysis. The results were expressed as means ± standard deviations (SD). One-way analyses of variance (ANOVA) followed by multiple comparison tests of means (Duncan’s new multiple range test) was used to compare the difference between the different treatments. The figures in the manuscript were generated using GraphPad Prism 8 and Adobe Photoshop CC.

## Results

### The effect of *C. morifolium-*maize intercropping on the agronomic traits and yield of *C. morifolium* and maize

**Figure 2:**
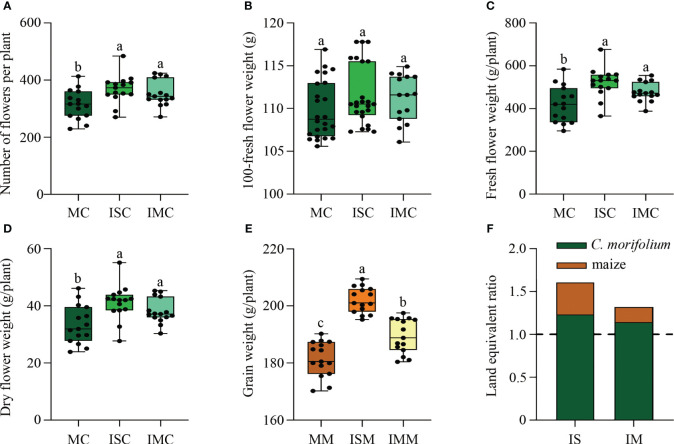
The effect of *C*. *morifolium-*maize intercropping on yield of *C*. *morifolium* and maize. *C*. *morifolium*: **(A)** Number of flowers per plant, **(B)** 100-fresh flower weight, **(C)** Fresh flower weight per plant, **(D)** Dry flower weight per plant. Maize: **(E)** Grain weight per plant. Data are shown as means ± standard deviations (n = 15). Boxes with different letters represent significant differences (*P* < 0.05). **(F)** The land equivalent ratio of different intercropping treatments.

Compared to MC, the agronomic traits of *C. morifolium* were increased in both ISC and IMC, with the number of second branches and flower inside diameter differing significantly in ISC (**Supplementary Table 1**). For the yield of *C. morifolium*, number of flowers per plant, fresh flower weight per plant, and dry flower weight per plant, were all highest in ISC and lowest in MC (**Figures 2A–D**). As for maize, grain weight per plant in ISM and IMM was significantly increased when compared to MM (**Figure 2E**). The land equivalent ratio is greater than 1, with the higheret in IS treatment, indicating that intercropping had a yield advantage (**Figure 2F**). These findings indicated that intercropping could promote the growth and development of *C. morifolium* and maize, especially in IS treatment.

### The effect of *C. morifolium-*maize intercropping on the active ingredients of *C. morifolium*

**Figure 3:**
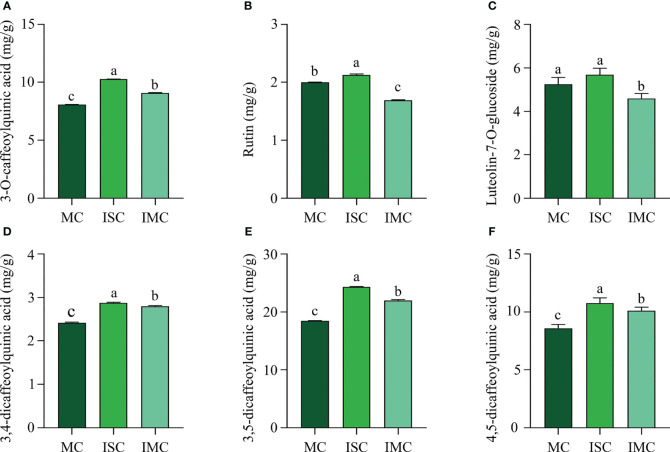
The effect of *C*. *morifolium-*maize intercropping on the active ingredients of *C*. *morifolium.* The content of **(A)** 3-O-caffeoylquinic acid, **(B)** Rutin, **(C)** Luteolin-7-O-glucoside, **(D)** 3,4-dicaffeoylquinic acid, **(E)** 3,5-dicaffeoylquinic acid, and **(F)** 4,5-dicaffeoylquinic acid. Data are shown as means ± standard deviations (n = 3). Columns with different letters represent significant differences (*P* < 0.05).

The content of primary active ingredients of plants are important quality characteristics. ISC contained the highest level of main active ingredients, including 3-O-caffeoylquinic acid (10.26 mg/kg), rutin (2.13 mg/kg), luteolin-7-O-glucoside (5.86 mg/kg), 3,4-dicaffeoylquinic acid (2.87 mg/kg), 3,5-dicaffeoylquinic acid (24.30 mg/kg) and 4,5-dicaffeoylquinic acid (10.76 mg/kg). Compared to MC, the content of 3-O-caffeoylquinic acid, 3,4-dicaffeoylquinic acid, 3,5-dicaffeoylquinic acid, and 4,5-dicaffeoylquinic acid, were significantly increased by 12.25%, 15.70%, 19.07% and 17.56%, respectively, while luteolin-7-O-glucoside and rutin were dramatically decreased by 12.40% and 15.50%, respectively in IMC (**Figures 3A–F**). In summary, intercropping could enhance quality of *C. morifolium*, particularly in IS treatment.

### The effect of *C. morifolium-*maize intercropping on soil physicochemical properties and enzyme activities of *C. morifolium* and maize

The physicochemical properties and enzyme activities of soil are closely related to plants. The contents of majority of soil physicochemical properties of *C. morifolium* were highest in IMC, containing SOM, NN, AvP, AvK, ExCa, ExMg, AvFe, AvMn, AvCu, AvZn, and AvB, reached 28.94 g/kg, 20.83 mg/kg, 37.36 mg/kg, 225.17 mg/kg, 411.63 mg/kg, 17.13 mg/kg, 82.09 mg/kg, 20.47 mg/kg, 2.61 mg/kg, 6.65 mg/kg, and 0.55 mg/kg, respectively. Contrasted with MC, ISC dramatically elevated the levels of SOM, TP, TK, AvP, AvFe, AvCu, and AvZn by 76.31%, 8.66%, 17.94%, 10.19%, 7.34%, 3.28%, and 2.06%, respectively, however, considerably lowered the contents of pH, AN, ExCa, and AvB by 2.21%, 2.61%, 13.33%, and 17.31%, respectively. As for maize, Compared to MM, pH value, the levels of SOM, TP, AN, AvP, ExCa, ExMg, AvFe, AvCu, and AvZn were greatly enhanced in ISM and IMM, the contents of SOM, TP, AvP, AvFe, AvCu, and AvZn were highest in IMM, and the levels of pH value, AN, ExCa, and ExMg peaked in ISM (**Table 1**).

**Table 1:** The effect of *C. morifolium-*maize intercropping on soil physicochemical properties of *C. morifolium* and maize.

Soil physicochemical properties	*C. morifolium*			maize		
	MC	ISC	IMC	MM	ISM	IMM
pH	7.25 ± 0.03a	7.09 ± 0.02b	6.50 ± 0.02c	6.30 ± 0.03c	6.50 ± 0.02b	6.92 ± 0.02a
SOM (g/kg)	12.79 ± 0.88c	22.55 ± 1.41b	28.94 ± 0.70a	10.93 ± 0.20c	20.11 ± 0.53b	21.27 ± 0.70a
TN (mg/kg)	167.33 ± 2.01a	168.04 ± 2.15a	97.84 ± 3.86b	192.69 ± 0.99c	203.02 ± 2.57b	235.06 ± 1.87a
TP (mg/kg)	166.71 ± 9.28b	181.14 ± 3.33a	108.03 ± 2.89c	105.15 ± 5.77b	120.54 ± 8.33a	116.69 ± 7.64ab
TK (g/kg)	30.55 ± 0.14c	36.03 ± 0.23a	34.58 ± 0.45b	32.32 ± 0.09a	24.43 ± 0.29c	28.38 ± 0.19b
AN (mg/kg)	17.98 ± 0.09a	17.51 ± 0.09b	16.93 ± 0.33c	8.70 ± 0.09c	11.58 ± 0.18b	15.20 ± 0.36a
NN (mg/kg)	10.08 ± 2.02b	11.42 ± 1.16b	16.80 ± 3.08a	30.05 ± 0.98b	29.48 ± 1.96b	38.55 ± 0.98a
AvP (mg/kg)	30.52 ± 2.06c	33.63 ± 0.45b	37.36 ± 0.65a	18.94 ± 5.42c	32.35 ± 3.10b	54.69 ± 6.88a
AvK (mg/kg)	167.35 ± 1.41b	164.87 ± 2.01b	225.17 ± 1.10a	129.53 ± 3.57b	105.58 ± 4.16c	156.69 ± 1.77a
ExCa (mg/kg)	362.62 ± 1.21b	314.29 ± 2.27c	411.63 ± 1.60a	357.27 ± 2.16c	492.34 ± 2.32a	420.50 ± 2.68b
ExMg (mg/kg)	16.01 ± 0.11b	15.93 ± 0.21b	17.13 ± 0.22a	15.68 ± 0.10b	17.24 ± 0.20a	16.98 ± 0.24a
AvFe (mg/kg)	48.10 ± 0.30c	51.63 ± 0.22b	82.09 ± 0.44a	35.40 ± 0.11c	44.44 ± 0.24b	95.54 ± 0.63a
AvMn (mg/kg)	20.47 ± 0.23a	20.44 ± 0.05a	19.70 ± 0.20b	19.60 ± 0.12b	19.35 ± 0.18b	20.49 ± 0.15a
AvCu (mg/kg)	1.83 ± 0.03c	1.89 ± 0.01b	2.61 ± 0.02a	1.18 ± 0.03c	1.34 ± 0.01b	2.05 ± 0.02a
AvZn (mg/kg)	5.82 ± 0.02c	5.94 ± 0.04b	6.65 ± 0.04a	4.13 ± 0.09c	4.78 ± 0.04b	5.91 ± 0.08a
AvB (mg/kg)	0.52 ± 0.03a	0.43 ± 0.03b	0.55 ± 0.02a	0.42 ± 0.02b	0.43 ± 0.02b	0.64 ± 0.03a

pH, soil organic matter (SOM), total nitrogen (TN), total phosphorus (TP), total potassium (TK), ammonium nitrogen (AN), nitrate nitrogen (NN), available phosphorus (AvP), available potassium (AvK), exchangeable calcium (ExCa), exchangeable magnesium (ExMg), available ferrum (AvFe), available manganese (AvMn), available cuprum (AvCu), available zinc (AvZn), available boron (AvB). Data are shown as means ± standard deviations (n = 3). Different letters represent significant differences (P < 0.05).

**Figure 4:**
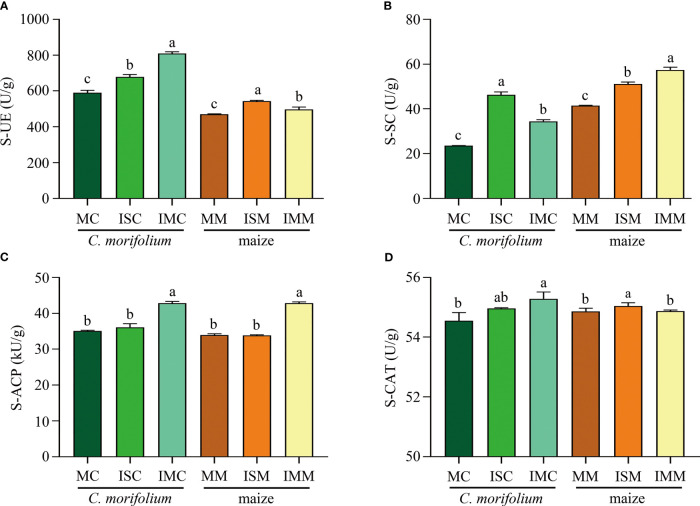
The effect of *C*. *morifolium-*maize intercropping on enzyme activities of *C*. *morifolium* and maize. **(A)** Soil urease (S-UE), **(B)** Soil sucrase (S-SC), **(C)** Soil acid phosphatase (S-ACP), **(D)** Soil catalase (S-CAT). Data are shown as means ± standard deviations (n = 3). Columns with different letters represent significant differences (*P* < 0.05).

For the soil enzyme activities of *C. morifolium*, S-UE, S-SC, S-ACP, and S-CAT were increased in ISC and IMC, IMC represented the highest S-UE activity (808.73 U/g), S-ACP activity (42.83 kU/g), and S-CAT activity (55.29 U/g), whereas ISC had the highest S-SC activity, reached 46.21 U/g. As for maize, Contrasted with MM, ISM significantly raised S-UE, S-SC, and S-CAT activities by 15.56%, 23.07%, and 0.33%, respectively; S-UE, S-SC, and S-ACP activities were dramatically increased by 5.84%, 38.63%, and 26.19%, respectively in IMM (**Figures 4A–D**). Generally, intercropping could promote most of soil physicochemical properties and enzyme activities, especially in IM treatment.

### The effect of *C. morifolium-*maize intercropping on rhizosphere soil microbial community characteristics of *C. morifolium* and maize

**Figure 5:**
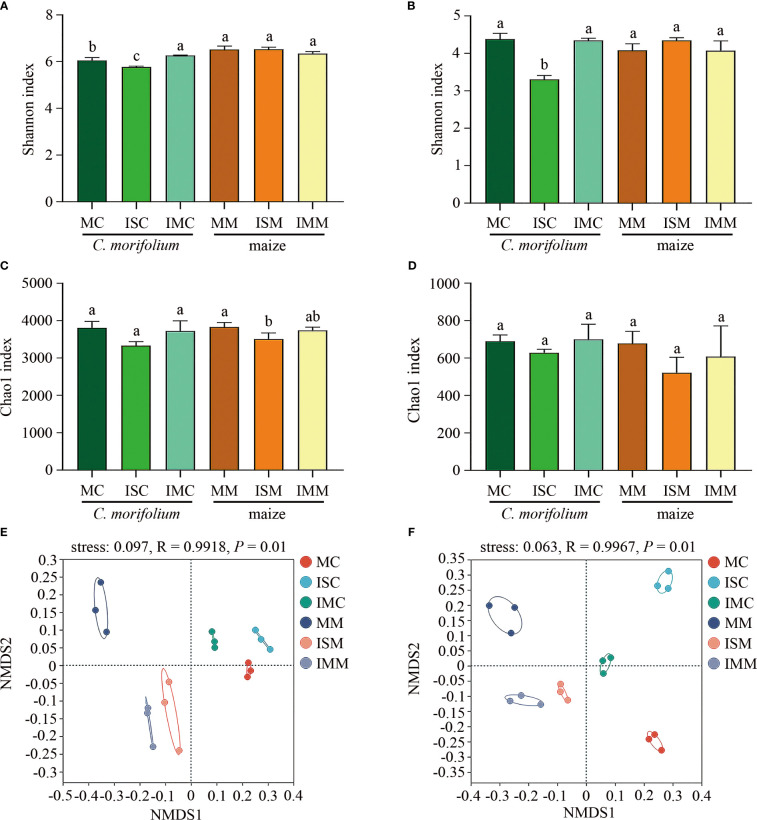
The effect of *C*. *morifolium-*maize intercropping on rhizosphere soil microbial community characteristics of *C*. *morifolium* and maize. The Shannon index at the OTU level: **(A)** Bacteria, **(B)** Fungi. The Chao1 index at the OTU level: **(C)** Bacteria, **(D)** Fungi. Data are shown as means ± standard deviations (n = 3). Columns with different letters represent significant differences (*p* < 0.05). NMDS analysis at the OTU level: **(E)** Bacteria, **(F)** Fungi.

Shannon and Chao1 indexes represent the diversity and richness of microbial communities, respectively. For the rhizosphere soil bacterial community, compared to MC, Shannon and Chao1 indexes were significantly reduced in ISC. Shannon index was markedly increased, while Chao1 index had no significant differences in IMC. Contrasted with MM, apart from ISM reduced Chao1 index, no considerable alterations were observed in Shannon and Chao1 indexes in ISM and IMM (**Figures 5A, C**). As for fungal community, Among the Shannon and Chao1 indexes of different treatments, only Shannon index between MC and ISC showed noteworthy distinctions (**Figures 5B, D**). The Shannon and Chao1 indexes demonstrated that intercropping induced greater changes in bacterial community than fungal community, particularly in IS treatment. Non-metric multidimensional scaling (NMDS) analysis revealed bacterial and fungal communities differed considerably between *C. morifolium* and maize, divided into two categories (by NMDS1). Moreover, ANOSIM analysis also showed soil bacterial (R = 0.9918, *P* = 0.001) and fungal (R = 0.9967, *P* = 0.001) community structures of different treatments had clear distinctions (**Figures 5E, F**).

### The effect of *C. morifolium-*maize intercropping on rhizosphere soil microbial community compositions of *C. morifolium* and maize

**Figure 6:**
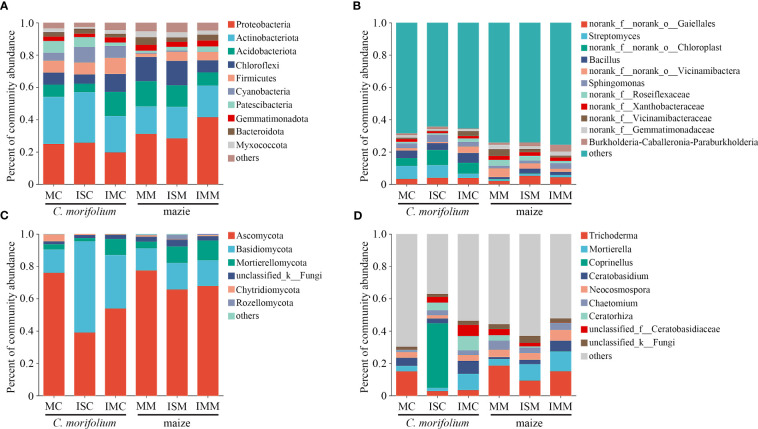
The effect of *C*. *morifolium*-maize intercropping on rhizosphere soil microbial community compositions of *C*. *morifolium* and maize. Percent of community abundance at the phylum level: **(A)** Bacteria, **(C)** Fungi. Percent of community abundance at the genus level: **(B)** Bacteria, **(D)** Fungi.

*C. morifolium* and maize rhizosphere soil microbial communities were all mainly composed of the bacterial phyla Proteobacteria (19.75%-41.50%), Actinobacteria (17.00%-31.34%), Acidobacteriota (5.21%-15.55%), and Chloroflexi (5.58%-15.06%) and the fungal phyla Ascomycota (39.11%-77.38%) and Basidiomycota (13.66%-56.25%) in different treatments (**Figures 6A, C**, **Supplementary Table 2**). However, the abundance of dominant microbial genera had an evident change. Compared to MC, *Sphingomonas*, *Burkholderia-Caballeronia-Paraburkholderia*, *Coprinellus*, *Chaetomium*, and *Ceratorhiza* were more enriched in ISC; *Bacillus*, *Sphingomonas*, *Ceratobasidium*, *Mortierella*, *Chaetomium*, and *Ceratorhiza* were more enriched in IMC. The proportion of *Streptomyces*, *Bacillus*, *Burkholderia-Caballeronia-Paraburkholderia*, *Mortierella*, and *Ceratobasidium* were all the lowest in MM, *Bacillus* increased most in ISM, and *Streptomyces*, *Sphingomonas*, *Burkholderia-Caballeronia-Paraburkholderia*, *Mortierella*, and *Ceratobasidium* peaked in IMM (**Figures 6B, D**, **Supplementary Table 3**).

### The effect of *C. morifolium-*maize intercropping on rhizosphere soil microbial co-occurrence patterns of *C. morifolium* and maize

**Figure 7:**
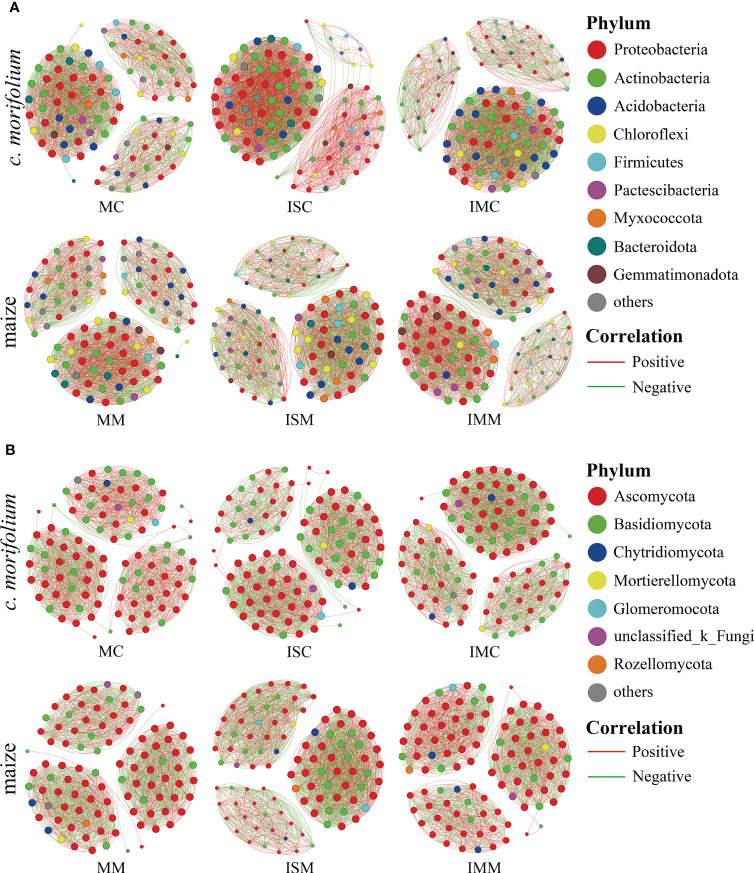
Co-occurrence network analysis of *C*. *morifolium* and maize rhizosphere soil microbial communities in different treatments. **(A)** Bacteria, **(B)** Fungi. Nodes indicate bacterial and fungal genera with relative abundance in the top 100; the size of each node is proportional to the number of connections, and the color represents the respective phylum. The links (lines between the nodes) indicate significant correlations (Spearman, |r| > 0.5, *P* < 0.05). Red and green lines represent positive and negative correlations, respectively.

Co-occurrence network analysis is commonly adopted to explore the interactions among microorganisms and uncover the differences between microbial communities. In both rhizosphere soil bacterial and fungal communities, compared to MC, the number of links, average degree, and graph density were increased in ISC and IMC; compared to MM, the number of links, average degree, and graph density were increased in ISM and IMM, suggesting that intercropping systems had more complex microbial networks (**Figures 7A, B**, **Supplementary Tables 4, 5**).

### The relationship among agronomic traits, yield, active ingredients, soil physicochemical properties, soil enzyme activities, and rhizosphere soil microbial communities of *C. morifolium* and maize

**Figure 8:**
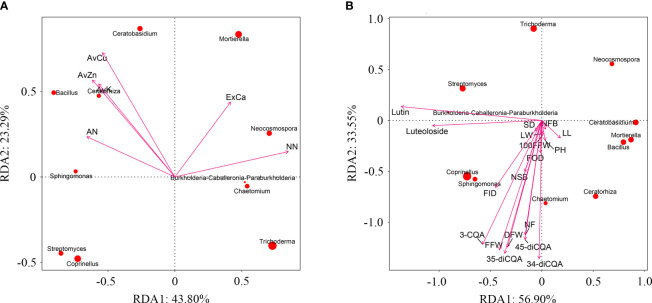
**(A)** Redundancy analysis of *C*. *morifolium* and maize soil environmental factors with dominant microorganisms at the genus level. Ammonium nitrogen (AN), nitrate nitrogen (NN), available potassium (AvK), exchangeable calcium (ExCa), available cuprum (AvCu), available zinc (AvZn). **(B)** Redundancy analysis of *C*. *morifolium* growth and quality indicators with dominant microorganisms at the genus level. Plant height (PH), stem diameter (SD), number of first branches (NFB), number of second branches (NSB), leaf length (LL), leaf width (LW), flower outside diameter (FOD), flower inside diameter (FID), number of flowers per plant (NF), 100-fresh flower weight (100FFW), fresh flower weight per plant (FFW), dry flower weight per plant (DFW), 3-O-caffeoylquinic acid (3-CQA), rutin, luteolin-7-O-glucoside (luteoloside), 3,4-dicaffeoylquinic acid (3,4-diCQA), 3,5-dicaffeoylquinic acid (3,5-diCQA), 4,5-dicaffeoylquinic acid (4,5-diCQA).

Use principal component analysis (PCA) to extract the main influencing factors based on all soil environmental indicators (soil physicochemical properties and enzyme activities) of *C. morifolium* and maize. The cumulative contribution of PC1 and PC2 amounted to 69.33% (**Supplementary Table 6**), the load of AN, AvK, AvCu, and AvZn, were larger in PC1, the load of NN and ExCa were larger in PC2, they were selected as representative soil environmental factors (**Supplementary Table 7**). Redundancy analysis (RDA) indicated that AN, AvK, AvCu, and AvZn were positively correlated with *Bacillus*, *Sphingomonas*, *Ceratobasidium*, and *Ceratorhiza*; NN and ExCa were positively correlated with *Burkholderia-Caballeronia-Paraburkholderia*, *Mortierella*, *Neocosmospora*, and *Chaetomium* (**Figure 8A**). Likewise, various agronomic traits, yield, and active ingredients of *C. morifolium* were positively correlated with *Bacillus*, *Sphingomonas*, *Burkholderia-Caballeronia-Paraburkholderia*, *Coprinellus*, *Chaetomium* and *Ceratorhiza* (**Figure 8B**). Moreover, plant height of *C. morifolium* was significantly positively correlated with AvZn, number of flowers per plant was significantly positively related to AvCu, rutin and luteolin-7-O-glucoside were significantly negatively related to AvK and ExCa (**Supplementary Table 8**).

## Discussion

In intercropping systems, the distance between crops has a great influence on soil microbial communities and physicochemical properties, and plants yield and quality. In this study, we designed two different *C. morifolium*-maize intercropping patterns, including *C. morifolium*-maize narrow-wide row planting (IS) and *C. morifolium*-maize middle row planting (IM). We found that the diversity and richness of the rhizosphere soil microbial communities in IS treatment were more variable than that in IM treatment (**Figures 5A–D**). We hypothesize that it may be because the spacing between intercropping crops affects the interactions between species (Li et al., 1999). Under IS treatment, the closer spacing between neighboring *C. morifolium* and maize caused more interaction. However, the promotion of the majority of soil physicochemical properties in IM treatment was greater than that in IS treatment (**Figure 4**). This may be due to the spacing between adjacent *C. morifolium* and maize was increased under IM treatment, which could reduce interspecific competition and lead to improved soil nutrition (Thorsted et al., 2006). In our study, *C. morifolium-*maize narrow-wide row planting had a higher improvement in *C. morifolium* yield and quality (**Figures 2**, **3**). Therefore, in intercropping systems, interspecific distance plays an important role in balancing nutrient utilization and microbial interaction.

Due to differences in crop species (Li and Wu, 2018) and root exudates (Jiang et al., 2022), intercropping impacts soil microbial communities. Previous studies have identified that intercropping could alter soil microbial characteristics and microbial community compositions (Sanguin et al., 2009; Bainard et al., 2012; Zhang et al., 2015). In our study, *C. morifolium* and maize rhizosphere soil bacterial and fungal networks in intercropping systems all exhibited higher average degrees and graph densities than monoculture, implying the interactions among microorganisms in intercropping systems were more active (**Figure 7**, **Supplementary Tables 4, 5**). In particular, intercropping with maize recruit a large number of beneficial microorganisms in the rhizosphere soil of *C. morifolium*, including *Bacillus*, *Sphingobium* and *Burkholderia-Caballeronia-Paraburkholderia*, *Chaetomium* and *Ceratorhiza* (**Figures 6B, D**). *Bacillus* was shown to inhibit *Verticillium dahliae* growth and induce systemic resistance in plants (Zhou et al., 2023). *Sphingomonas* had plant growth-promoting effects, which attributed to the ability to produce plant growth hormones such as gibberellins and indole acetic acid (Asaf et al., 2020; Jin et al., 2023). *Burkholderia-Caballeronia-Paraburkholderia* had the ability to induce systemic resistance against grey mold disease, and contained genes involved in plant growth promotion and biocontrol (Esmaeel et al., 2020). *Chaetomium* produced an array of antibiotics and secondary metabolites, thereby preventing pathogen entry and colonization of plant tissues and reducing the harmful microbial population (Seethapathy et al., 2023). *Ceratorhiza* produced plant growth regulators to stimulate significantly plant root development (Grönberg et al., 2006). They could promote plant growth and development by producing auxin and inhibiting disease. Therefore, the increased abundance of them in intercropping systems, may be one of the reasons for the high yield and quality of *C. morifolium* (**Figure 8B**).

It has been suggested that crop roots interact extensively with soil microorganisms to promote plant growth and development by affecting soil nutrient availability (Richardson et al., 2009). We also studied the relationship between soil physicochemical properties and rhizosphere microbial microorganisms of *C. morifolium* and maize. We found that *C. morifolium-*maize intercropping systems showed an enrichment of several beneficial bacterial genera in regulating soil condition, including *Sphingomonas*, *Bacillus*, and *Burkholderia-Caballeronia-Paraburkholderia* (**Figure 6B**). *Sphingomonas* was one of nitrogen-fixing bacteria, which could increase nitrogen utilization efficiency with significant impacts on nutrient transformation, thereby promoting sustainable growth and productivity of plants (Videira et al., 2009; Jalal et al., 2022). *Bacillus* has been documented to solubilize potassium for improving crop production (Basak and Biswas, 2009). *Bacillus* also had the ability to solubilize zinc to support plants growth and yield (Khande et al., 2017; Masood et al., 2022). *Burkholderia-Caballeronia-Paraburkholderia* was known to fix nitrogen (Tapia-García et al., 2020), and widely promoted plant growth through nitrogen-dependent mechanisms (Madhaiyan et al., 2021). Moreover, these enriched microbes were positively correlated with AN, NN, AvK, and AvZn in soil through redundancy analysis (**Figure 8A**). In conclusion, intercropping could increase the abundance of soil beneficial microorganisms, which could promote soil conditions, thus improving the overall growing environment and ultimately improving the growth and quality of *C. morifolium.*

## Conclusion

We examined the advantages of intercropping *C. morifolium* with maize. The results indicated that intercropping increased the agronomic traits, yield, and active ingredients of *C. morifolium*, especially in the IS treatment. Intercropping could also promote the soil physicochemical properties and enzyme activities, and change rhizosphere soil microbial communities of *C. morifolium* and maize. Moreover, intercropping could recruit a large number of beneficial bacteria enrich in the soil, including *Bacillus*, *Sphingobium* and *Burkholderia-Caballeronia-Paraburkholderia*, *Chaetomium*, and *Ceratorhiza*, which could increase the content of AN, NN, AvK, ExCa, AvCu, AvZn and other soil nutrients, improve the overall growing environment, and achieved the growth and improvement of *C. morifolium*. Overall, this study revealed that intercropping could produce more beneficial effects than monoculture, especially in *C. morifolium-*maize narrow-wide row planting patterns. Therefore, this pattern is worth popularizing in *C. morifolium* production.

## Data availability statement

The datasets presented in this study can be found in online repositories. The names of the repository/repositories and accession number(s) can be found in the article/**Supplementary Material**.

## Author contributions

ZL: Conceptualization, Formal analysis, Validation, Writing – original draft. QC: Formal analysis, Writing – review & editing. JL: Software, Writing – review & editing. LW: Methodology, Writing – review & editing. JW: Data curation, Writing – review & editing. XW: Investigation, Writing – review & editing. QL: Resources, Writing – review & editing. YM: Visualization, Writing – review & editing. DL: Conceptualization, Funding acquisition, Project administration, Supervision, Writing – review & editing.
