# Supplementary material for: Influence of Chrysanthemum morifolium-maize intercropping pattern on yield, quality, soil condition, and rhizosphere soil microbial communities of C. morifolium
Source: Front Plant Sci. 2024 Apr 24;15:1383477. doi: 10.3389/fpls.2024.1383477 (PMC11076860; doi:10.3389/fpls.2024.1383477)
Supplement: Supplementary file 1 [file Table_1.docx]

Supplementary Material

**Supplementary Table 1.** The effect of *C. morifolium*-maize intercropping on agronomic traits of *C. morifolium*. Data are shown as means ± standard deviations (n = 15). Different letters represent significant differences (*P* < 0.05).

| Agronomic trait | MC | ISC | IMC |
| --- | --- | --- | --- |
| Plant height (cm) | 50.20 ± 5.76a | 52.87 ± 4.87a | 52.73 ± 4.93a |
| Stem diameter (mm) | 11.84 ± 1.56a | 12.34 ± 1.48a | 12.07 ± 1.86a |
| Number of first branches | 5.60 ± 1.72a | 5.87 ± 1.06a | 5.87 ± 0.83a |
| Number of second branches | 17.27 ± 2.81b | 19.93 ± 3.26a | 18.73 ± 3.47ab |
| Leaf length (cm) | 8.50 ± 0.62a | 8.66 ± 0.65a | 8.88 ± 0.39a |
| Leaf width (cm) | 5.65 ± 0.53a | 5.87 ± 0.56a | 5.85 ± 0.43a |
| Flower outside diameter (mm) | 44.60 ± 6.77a | 48.24 ± 6.88a | 47.26 ± 7.79a |
| Flower inside diameter (mm) | 11.16 ± 1.25b | 12.42 ± 0.75a | 11.57 ± 1.55ab |

**Supplementary Table 2.** Comparison of the relative abundance of dominant phylum of *C. morifolium* and maize rhizosphere soil bacterial and fungal communities in different treatments. Data are shown as means ± standard deviations (n = 3). Different letters represent significant differences (*P* < 0.05).

|  | Phylum | *C. morifolium* | | |  | maize | | |
| --- | --- | --- | --- | --- | --- | --- | --- | --- |
|  |  | MC | ISC | IMC |  | MM | ISM | IMM |
| Bacteria | Proteobacteria (%) | 24.99 ± 1.43a | 25.79 ± 3.51a | 19.75 ± 0.75b |  | 31.21 ± 2.36b | 28.51 ± 4.38b | 41.50 ± 3.63a |
|  | Actinobacteriota (%) | 29.12 ± 0.92a | 31.34 ± 4.78a | 22.57 ± 1.80b |  | 17.00 ± 2.32a | 19.48 ± 3.95a | 19.59 ± 1.31a |
|  | Acidobacteriota (%) | 7.65 ± 1.15b | 5.21 ± 2.51b | 14.94 ± 2.17a |  | 15.55 ± 0.56a | 13.33 ± 0.68a | 8.23 ± 2.54b |
|  | Chloroflexi (%) | 7.27 ± 0.74b | 5.58 ± 1.71b | 11.02 ± 0.55a |  | 15.01 ± 1.60a | 15.06 ± 1.55a | 7.39 ± 1.41b |
|  | Firmicutes (%) | 7.52 ± 0.48b | 7.47 ± 0.81b | 10.08 ± 0.59a |  | 2.12 ± 0.33b | 5.73 ± 0.60a | 5.24 ± 0.67a |
|  | Cyanobacteria (%) | 4.91 ± 1.81a | 9.49 ± 4.19a | 7.19 ± 0.57a |  | 0.57 ± 0.17a | 0.73 ± 0.06a | 0.19 ± 0.02b |
|  | Patescibacteria (%) | 7.14 ± 0.48a | 6.20 ± 0.25b | 2.15 ± 0.14c |  | 1.22 ± 0.13b | 2.15 ± 0.76ab | 3.14 ± 1.07a |
|  | Gemmatimonadota (%) | 2.86 ± 0.19a | 2.24 ± 0.31b | 3.29 ± 0.18a |  | 3.73 ± 0.81a | 3.25 ± 0.38a | 3.84 ± 0.40a |
|  | Bacteroidota (%) | 2.66 ± 0.16a | 2.88 ± 0.23a | 2.18 ± 0.25b |  | 4.50 ± 0.82a | 2.61 ± 0.45b | 3.55 ± 0.82ab |
|  | Myxococcota (%) | 2.39 ± 0.32a | 1.21 ± 0.15b | 2.25 ± 0.15a |  | 3.79 ± 0.88a | 3.20 ± 0.40a | 2.56 ± 0.50a |
| Fungi | Ascomycota (%) | 75.88 ± 6.01a | 39.11 ± 0.77c | 53.90 ± 2.94b |  | 77.38 ± 3.72a | 65.66 ± 5.66b | 67.81 ± 4.24b |
|  | Basidiomycota (%) | 14.37 ± 0.43c | 56.25 ± 1.33a | 33.01 ± 1.28b |  | 13.66 ± 2.95a | 16.38 ± 4.92a | 15.84 ± 5.76a |
|  | Mortierellomycota (%) | 3.24 ± 0.49b | 2.01 ± 0.30b | 9.94 ± 2.27a |  | 4.06 ± 1.07b | 10.15 ± 2.37a | 12.24 ± 2.55a |
|  | unclassified_k__Fungi (%) | 1.82 ± 0.41a | 1.82 ± 0.35a | 2.47 ± 0.53a |  | 3.02 ± 1.19a | 4.24 ± 1.86a | 2.55 ± 0.83a |
|  | Chytridiomycota (%) | 4.30 ± 7.16a | 0.67 ± 0.04a | 0.43 ± 0.16a |  | 0.87 ± 0.79a | 0.46 ± 0.22a | 0.65 ± 0.55a |
|  | Rozellomycota (%) | 0.05 ± 0.01a | 0.03 ± 0.02ab | 0.01 ± 0.01b |  | 0.47 ± 0.29b | 2.51 ± 1.49a | 0.74 ± 0.28ab |

**Supplementary Table 3.** Comparison of the relative abundance of dominant genus of *C. morifolium* and maize rhizosphere soil bacterial and fungal communities in different treatments. Data are shown as means ± standard deviations (n = 3). Different letters represent significant differences (*P* < 0.05).

|  | Genus | *C. morifolium* | | |  | maize | | |
| --- | --- | --- | --- | --- | --- | --- | --- | --- |
|  |  | MC | ISC | IMC |  | MM | ISM | IMM |
| Bacteria | norank_f__norank_o__Gaiellales (%) | 3.37 ± 0.30b | 4.15 ± 0.50ab | 4.03 ± 0.26b |  | 2.07 ± 0.26b | 5.30 ± 0.85a | 4.40 ± 0.58a |
|  | Streptomyces (%) | 8.13 ± 0.81a | 7.80 ± 0.81a | 2.61 ± 0.10b |  | 1.01 ± 0.36a | 1.09 ± 0.18a | 1.30 ± 0.02a |
|  | norank_f__norank_o__Chloroplast (%) | 4.78 ± 1.81a | 9.27 ± 4.11a | 6.66 ± 0.39a |  | 0.30 ± 0.12a | 0.41 ± 0.12a | 0.08 ± 0.02b |
|  | Bacillus (%) | 4.63 ± 0.38b | 4.23 ± 0.71b | 6.09 ± 0.17a |  | 1.08 ± 0.08c | 2.85 ± 0.36a | 1.96 ± 0.28b |
|  | norank_f__norank_o__Vicinamibacterales (%) | 1.59 ± 0.29b | 0.80 ± 0.69b | 4.03 ± 1.36a |  | 5.50 ± 0.32a | 3.40 ± 0.38b | 1.91 ± 1.01c |
|  | Sphingomonas (%) | 2.90 ± 0.24b | 4.39 ± 0.66a | 2.97 ± 0.20b |  | 1.54 ± 0.34b | 0.90 ± 0.65b | 3.21 ± 1.27a |
|  | norank_f__Roseiflexaceae (%) | 1.00 ± 0.06b | 0.98 ± 0.06b | 1.98 ± 0.15a |  | 3.54 ± 0.28a | 2.93 ± 0.43a | 1.36 ± 0.26b |
|  | norank_f__Xanthobacteraceae (%) | 1.69 ± 0.18a | 1.20 ± 0.15b | 1.57 ± 0.21ab |  | 2.52 ± 0.64a | 2.33 ± 0.30a | 2.21 ± 0.19a |
|  | norank_f__Vicinamibacteraceae (%) | 0.88 ± 0.17a | 0.40 ± 0.38a | 2.91 ± 1.29b |  | 4.09 ± 0.29a | 1.87 ± 0.37b | 1.13 ± 0.71b |
|  | norank_f__Gemmatimonadaceae (%) | 1.52 ± 0.11a | 0.97 ± 0.12b | 1.40 ± 0.04a |  | 2.69 ± 0.67a | 1.83 ± 0.29a | 2.55 ± 0.23a |
|  | Burkholderia-Caballeronia-Paraburkholderia (%) | 1.20 ± 0.12b | 1.58 ± 0.20a | 0.28 ± 0.03c |  | 1.43 ± 1.09a | 2.26 ± 2.23a | 4.12 ± 0.79a |
| Fungi | Trichoderma (%) | 15.08 ± 0.72a | 2.89 ± 0.52b | 3.58 ± 0.41b |  | 18.67 ± 7.72a | 9.41 ± 3.03a | 15.11 ± 5.59a |
|  | Mortierella (%) | 3.23 ± 0.49b | 2.00 ± 0.30b | 9.87 ± 2.25a |  | 4.05 ± 1.07b | 10.14 ± 2.37a | 12.22 ± 2.54a |
|  | Coprinellus (%) | 0.28 ± 0.07b | 39.82 ± 1.38a | 0.10 ± 0.05b |  | 0.03 ± 0.01a | 0.02 ± 0.01a | 0.15 ± 0.13a |
|  | Ceratobasidium (%) | 4.81 ± 0.42b | 2.95 ± 0.25c | 8.01 ± 0.46a |  | 1.12 ± 0.16b | 2.60 ± 1.07a | 6.51 ± 3.10a |
|  | Neocosmospora (%) | 3.69 ± 0.39a | 2.11 ± 0.39b | 3.64 ± 0.18a |  | 4.69 ± 0.93a | 4.28 ± 0.28a | 6.74 ± 3.76a |
|  | Chaetomium (%) | 1.21 ± 0.52b | 2.92 ± 0.55a | 2.82 ± 1.04a |  | 5.46 ± 3.34a | 3.26 ± 0.56a | 4.10 ± 0.71a |
|  | Ceratorhiza (%) | 0.01 ± 0.01c | 4.78 ± 0.62b | 8.84 ± 0.20a |  | 3.29 ± 1.22a | 0.77 ± 0.40b | 0.00 ± 0.01b |
|  | unclassified_f__Ceratobasidiaceae (%) | 0.16 ± 0.07c | 3.61 ± 0.30b | 6.96 ± 0.93a |  | 3.85 ± 1.40a | 2.31 ± 0.85a | 0.36 ± 0.16b |
|  | unclassified_k__Fungi (%) | 1.82 ± 0.41a | 1.82 ± 0.35a | 2.47 ± 0.53a |  | 3.02 ± 1.19a | 4.24 ± 1.86a | 2.55 ± 0.83a |

**Supplementary Table 4.** The co-occurrence network analysis topological properties of *C. morifolium* and maize rhizosphere soil bacterial communities in different treatments.

| Network indexes | *C. morifolium* | | |  | maize | | |
| --- | --- | --- | --- | --- | --- | --- | --- |
|  | MC | ISC | IMC |  | MM | ISM | IMM |
| Nodes | 98 | 97 | 98 |  | 100 | 100 | 1100 |
| Links | 1616 | 2184 | 1894 |  | 1635 | 1711 | 1751 |
| Positive links | 891 | 1410 | 1052 |  | 911 | 868 | 997 |
| Negative links | 725 | 774 | 842 |  | 724 | 843 | 754 |
| Average degree | 32.980 | 45.031 | 38.563 |  | 32.700 | 34.220 | 35.020 |
| Network diameter | 1 | 1 | 1 |  | 1 | 1 | 1 |
| Average path length | 1 | 1 | 1 |  | 1 | 1 | 1 |
| Graph density | 0.340 | 0.469 | 0.398 |  | 0.330 | 0.346 | 0.354 |
| Modularity | 0.550 | 0.313 | 0.399 |  | 0.594 | 0.590 | 0.578 |
| Average clustering coefficient | 1 | 1 | 1 |  | 1 | 1 | 1 |

**Supplementary Table 5.** The co-occurrence network analysis topological properties of *C. morifolium* and maize rhizosphere soil fungal communities in different treatments.

| Network indexes | *C. morifolium* | | |  | maize | | |
| --- | --- | --- | --- | --- | --- | --- | --- |
|  | MC | ISC | IMC |  | MM | ISM | IMM |
| Nodes | 100 | 100 | 99 |  | 99 | 99 | 99 |
| Links | 1377 | 1428 | 1593 |  | 1469 | 1695 | 1510 |
| Positive links | 805 | 751 | 807 |  | 742 | 852 | 837 |
| Negative links | 572 | 677 | 786 |  | 727 | 843 | 673 |
| Average degree | 27.540 | 28.560 | 32.182 |  | 29.677 | 34.242 | 30.505 |
| Network diameter | 1 | 1 | 1 |  | 1 | 1 | 1 |
| Average path length | 1 | 1 | 1 |  | 1 | 1 | 1 |
| Graph density | 0.278 | 0.288 | 0.328 |  | 0.303 | 0.349 | 0.311 |
| Modularity | 0.666 | 0.627 | 0.601 |  | 0.659 | 0.581 | 0.653 |
| Average clustering coefficient | 1 | 1 | 1 |  | 1 | 1 | 1 |

**Supplementary Table 6.** Table of total variance of principal component analysis (PCA) of *C. morifolium* and maize soil physicochemical properties and enzyme activities.

| Principal components | Initial eigenvalues | | |  | Extraction Sums of Squared Loadings | | |
| --- | --- | --- | --- | --- | --- | --- | --- |
|  | Total | Variance percentage/% | Cumulative percentage/% |  | Total | Variance percentage/% | Cumulative percentage/% |
| 1 | 8.146 | 40.728 | 40.728 |  | 8.146 | 40.728 | 40.728 |
| 2 | 5.720 | 28.602 | 69.330 |  | 5.720 | 28.602 | 69.330 |
| 3 | 3.620 | 18.100 | 87.430 |  | 3.620 | 18.100 | 87.430 |
| 4 | 1.386 | 6.932 | 94.362 |  | 1.386 | 6.932 | 94.362 |
| 5 | 1.128 | 5.638 | 100.000 |  | 1.128 | 5.638 | 100.000 |
| 6 | 5.52E-16 | 2.76E-15 | 100.000 |  |  |  |  |
| 7 | 4.43E-16 | 2.21E-15 | 100.000 |  |  |  |  |
| 8 | 3.45E-16 | 1.72E-15 | 100.000 |  |  |  |  |
| 9 | 2.14E-16 | 1.07E-15 | 100.000 |  |  |  |  |
| 10 | 1.90E-16 | 9.50E-16 | 100.000 |  |  |  |  |
| 11 | 1.77E-16 | 8.87E-16 | 100.000 |  |  |  |  |
| 12 | 1.27E-16 | 6.35E-16 | 100.000 |  |  |  |  |
| 13 | 5.81E-17 | 2.91E-16 | 100.000 |  |  |  |  |
| 14 | -1.85E-17 | -9.26E-17 | 100.000 |  |  |  |  |
| 15 | -7.10E-17 | -3.55E-16 | 100.000 |  |  |  |  |
| 16 | -9.90E-17 | -4.95E-16 | 100.000 |  |  |  |  |
| 17 | -1.69E-16 | -8.45E-16 | 100.000 |  |  |  |  |
| 18 | -1.95E-16 | -9.74E-16 | 100.000 |  |  |  |  |
| 19 | -3.37E-16 | -1.68E-15 | 100.000 |  |  |  |  |
| 20 | -4.09E-16 | -2.05E-15 | 100.000 |  |  |  |  |

**Supplementary Table 7.** Table of matrix components of principal component analysis (PCA) of *C. morifolium* and maize soil physicochemical properties and enzyme activities. pH, soil organic matter (SOM), total nitrogen (TN), total phosphorus (TP), total potassium (TK), ammonium nitrogen (AN), nitrate nitrogen (NN), available phosphorus (AvP), available potassium (AvK), exchangeable calcium (ExCa), exchangeable magnesium (ExMg), available ferrum (AvFe), available manganese (AvMn), available cuprum (AvCu), available zinc (AvZn), available boron (AvB), soil urease (S-UE), soil sucrase (S-SC), soil acid phosphatase (S-ACP), soil catalase (S-CAT).

| Indicators | Principal components | | | | |
| --- | --- | --- | --- | --- | --- |
|  | 1 | 2 | 3 | 4 | 5 |
| pH | 0.388 | -0.535 | 0.697 | 0.276 | 0.044 |
| SOM | 0.770 | 0.399 | -0.297 | 0.179 | 0.358 |
| TN | -0.581 | 0.335 | 0.719 | -0.040 | 0.176 |
| TP | 0.175 | -0.779 | 0.359 | 0.369 | 0.312 |
| TK | 0.473 | -0.592 | -0.321 | -0.502 | 0.266 |
| AN | 0.843 | -0.381 | 0.263 | 0.274 | 0.002 |
| NN | -0.395 | 0.830 | 0.256 | -0.299 | 0.024 |
| AvP | 0.606 | 0.544 | 0.566 | 0.045 | 0.118 |
| AvK | 0.909 | -0.173 | -0.243 | -0.227 | -0.183 |
| ExCa | -0.124 | 0.826 | -0.098 | 0.491 | -0.229 |
| ExMg | 0.360 | 0.822 | -0.098 | 0.426 | -0.066 |
| AvFe | 0.759 | 0.546 | 0.289 | -0.204 | -0.034 |
| AvMn | 0.449 | -0.429 | 0.767 | -0.135 | 0.093 |
| AvCu | 0.990 | 0.072 | -0.064 | -0.053 | -0.083 |
| AvZn | 0.987 | -0.061 | 0.083 | 0.124 | -0.009 |
| AvB | 0.643 | 0.444 | 0.471 | -0.187 | -0.363 |
| S-UE | 0.795 | -0.208 | -0.532 | 0.185 | 0.090 |
| S-SC | -0.189 | 0.686 | 0.291 | -0.076 | 0.635 |
| S-NP | 0.814 | 0.475 | 0.110 | -0.310 | -0.055 |
| S-CAT | 0.361 | 0.510 | -0.693 | -0.003 | 0.361 |

**Supplementary Table 8.** Spearman correlation analysis of *C. morifolium* soil environmental factors with growth and quality indicators. *C. morifolium* soil environmental factors: ammonium nitrogen (AN), nitrate nitrogen (NN), available potassium (AvK), exchangeable calcium (ExCa), available cuprum (AvCu), available zinc (AvZn). *C. morifolium* growth and quality indicators: plant height (PH), stem diameter (SD), number of first branches (NFB), number of second branches (NSB), leaf length (LL), leaf width (LW), flower outside diameter (FOD), flower inside diameter (FID), number of flowers per plant (NF), 100-fresh flower weight (100FFW), fresh flower weight per plant (FFW), dry flower weight per plant (DFW), 3-O-caffeoylquinic acid (3-CQA), rutin, luteolin-7-O-glucoside (luteoloside), 3,4-dicaffeoylquinic acid (3,4-diCQA), 3,5-dicaffeoylquinic acid (3,5-diCQA), 4,5-dicaffeoylquinic acid (4,5-diCQA) (* P < 0.05, ** P < 0.01, *** P < 0.001).

|  | AN | NN | AvK | ExCa | AvCu | AvZn |
| --- | --- | --- | --- | --- | --- | --- |
| PH | -0.378 | 0.162 | 0.317 | 0.217 | 0.483 | 0.717* |
| SD | -0.235 | 0.264 | 0.067 | 0.033 | 0.133 | 0.150 |
| NFB | -0.194 | 0.246 | 0.201 | -0.140 | 0.271 | 0.219 |
| NSB | -0.291 | 0.231 | -0.444 | -0.159 | 0.201 | 0.117 |
| LL | -0.420 | 0.230 | 0.150 | 0.067 | 0.467 | 0.250 |
| LW | -0.316 | 0.094 | -0.075 | -0.067 | 0.351 | 0.310 |
| FOD | -0.496 | 0.264 | -0.067 | -0.267 | 0.533 | 0.267 |
| FID | -0.261 | -0.043 | -0.567 | -0.550 | 0.233 | 0.217 |
| NF | -0.639 | 0.519 | -0.217 | -0.250 | 0.683* | 0.567 |
| 100FFW | -0.269 | 0.221 | 0.200 | -0.200 | 0.300 | 0.033 |
| FFW | -0.462 | 0.281 | -0.350 | -0.517 | 0.533 | 0.450 |
| DFW | -0.487 | 0.255 | -0.333 | -0.550 | 0.533 | 0.417 |
| 3-CQA | -0.462 | 0.247 | -0.283 | -0.433 | 0.417 | 0.433 |
| Lutin | 0.437 | -0.621 | -0.900*** | -0.867** | -0.517 | -0.483 |
| Luteoloside | 0.546 | -0.630 | -0.817** | -0.783* | -0.600 | -0.467 |
| 3,4-diCQA | -0.513 | 0.264 | -0.300 | -0.483 | 0.450 | 0.383 |
| 3,5-diCQA | -0.471 | 0.306 | -0.367 | -0.450 | 0.417 | 0.383 |
| 4,5-diCQA | -0.479 | 0.281 | -0.183 | -0.300 | 0.533 | 0.600 |
